# Effects of message framing and risk perception on health communication for optimum cardiovascular disease primary prevention: a protocol for a multicenter randomized controlled study

**DOI:** 10.3389/fpubh.2024.1308745

**Published:** 2024-03-14

**Authors:** Zhiting Guo, Qunhua Wu, Xiaomei Wang, Yuehua Dai, Yajun Ma, YunJing Qiu, Yuping Zhang, Xuyang Wang, Jingfen Jin

**Affiliations:** ^1^Nursing Department, The Second Affiliated Hospital of Zhejiang University School of Medicine (SAHZU), Hangzhou, China; ^2^Faculty of Nursing, Zhejiang University School of Medicine, Hangzhou, China; ^3^Referral Office, The People’s No.3 Hospital of Hangzhou Xiaoshan, Hangzhou, China; ^4^School of Media, Hangzhou City University, Hangzhou, China; ^5^Office of Chronic Disease Management, Nanxing Community Health Service Center, Hangzhou, China; ^6^School of Nursing and Midwifery, Faculty of Health, University of Technology Sydney, Sydney, NSW, Australia; ^7^Key Laboratory of the Diagnosis and Treatment of Severe Trauma and Burn of Zhejiang Province, Hangzhou, China

**Keywords:** cardiovascular disease, primary prevention, message framing, health communication, risk perception, randomized controlled trial

## Abstract

**Background:**

Although several guidelines for cardiovascular disease (CVD) management have highlighted the significance of primary prevention, the execution and adherence to lifestyle modifications and preventive medication interventions are insufficient in everyday clinical practice. The utilization of effective risk communication can assist individuals in shaping their perception of CVD risk, motivating them to make lifestyle changes, and increasing their willingness to engage with preventive medication, ultimately reducing their CVD risks and potential future events. However, there is limited evidence available regarding the optimal format and content of CVD risk communication.

**Objective:**

The pilot study aims to elucidate the most effective risk communication strategy, utilizing message framing (gain-framed, loss-framed, or no-framed), for distinct subgroups of risk perception (under-perceived, over-perceived, and correctly-perceived CVD risk) through a multi-center randomized controlled trial design.

**Methods:**

A multi-center 3 × 3 factorial, observer-blinded experimental design was conducted. The participants will be assigned into three message-framing arms randomly in a 1:1:1 ratio and will receive an 8-week intervention online. Participants are aged 20–80 years old and have a 10-year risk of absolute CVD risk of at least 5% (moderate risk or above). We plan to enroll 240 participants based on the sample calculation. The primary outcome is the CVD prevention behaviors and CVD absolute risk value. Data collection will occur at baseline, post-intervention, and 3-month follow-up.

**Discussion:**

This experimental study will expect to determine the optimal matching strategy between risk perception subgroups and risk information format, and it has the potential to offer health providers in community or clinic settings a dependable and efficient health communication information template for conducting CVD risk management.

**Clinical trial registration**: https://www.chictr.org.cn/bin/project/edit?pid=207811, ChiCTR2300076337.

## 1 Introduction

Cardiovascular disease (CVD) is the leading cause of death in China, with two out of every five deaths attributed to CVD (1, 2). Over 95% of all CVD deaths are attributable to IHD, stroke, hypertensive heart disease, cardiomyopathy, rheumatic heart disease, and atrial fibrillation (3). Currently, China faces a dual challenge with an aging population and the persisting prevalence of cardiometabolic risk factors, particularly the rates of hypertension, dyslipidemia, and diabetes have reached alarming levels (4), and the burden of CVD is projected to escalate in the future. Therefore, it is imperative to devise effective and robust strategies to raise awareness, improve treatment, and enhance control rates for these conditions.

Lifestyle management and risk factor control form the fundamental to both primary and secondary CVD prevention, as underscored by all major CVD management guidelines (5, 6). Despite this emphasis, implementation and adherence to lifestyle modifications and preventive medication interventions remain inadequate in daily practice (7). CVD risk, which is the likelihood of experiencing a cardiovascular event over a specific time frame (e.g., 10 years), is calculated by mathematically combining multiple predictors (8). Healthcare providers use this information to guide prevention strategies and interventions aimed at reducing CVD risk, such as lifestyle modifications and medication. However, a large number of individuals with risk factors remain unaware of their CVD risk, its implications, and the rationale for medication and lifestyle modification (9). Additionally, lifestyle modification interventions often neglect the individual’s preferences, perceptions, and characteristics, which could potentially contribute to suboptimal risk factor management.

Risk perception serves as a cognitive process influencing health behavior and has been regarded as a critical element in various socio-cognitive theories of health behavior, including the Health Belief Model (10), Protection Motivation Theory (11), and Risk Perception Attitude Framework (12) (Supplementary Table S1). Notably, risk perception has also been confirmed to be a motivational determinant that plays a key role in participation and adherence in health-promoting behaviors in empirical researches (13–15). More specify, CVD risk perception, the belief that the individual is vulnerable to develop CVD (16), could affect health behavior change and maintenance (17). However, most people hold inaccurate perception toward their risk to develop CVD when compared with the objective calculated CVD risk (18), our previous research founded that only 30.1% participants accurately perceived their CVD risk (19). These perception bias may lead to diminished motivation, thereby hindering the exertion of requisite effort to mitigate their risk (20). To address this issue, an educational program was designed and proven to effectively improve risk perception for community residents (21) or risk populations (22) in several studies. However, a ceiling effect was revealed for smoker and obesity subgroups. These findings revealed that future campaigns should target risk populations that remarkably hold risk misperceptions.

Prospect theory, developed by Daniel Kahneman and Amos Tversky in 1979, is a psychological theory that describes how people make decisions under uncertainty (23). Prospect theory suggests that individuals weigh potential health outcomes differently depending on whether they perceive them as gains or losses which can affect health-related decision-making (24). Risk communication plays a pivotal role in shared decision-making processes, informing individuals about their CVD risk level and options for risk reduction, thus correcting inappropriate risk perception (25). Previous research has shown that both ‘what’ is communicated and “how” it is conveyed significantly influence an individual’s understanding of CVD risk levels, empowerment, and autonomy (26, 27). Effective risk communication can guide individuals in shaping their perceived CVD risk, motivating them toward lifestyle alterations, and enhancing their willingness to engage with preventive medication, thereby decreasing their CVD risks and potential future events (28). Although current published guidelines suggest discussing the individual CVD risk with patients, there is limited guidance on the appropriate format and optimal presentation strategies (29). Communicating risk is challenging, and the quality of consultation depends on the interpretation and discussion between healthcare practitioners and individuals at risk (30). However, interpretation has consistently been insufficient, and the delivery of information has been inconsistent. Both parties struggle to comprehend CVD risk, and some practitioners lack confidence in explaining risk scores, resulting in inadequate recall of individual CVD risk, confusion, and misunderstandings (31). Given the limited duration of practitioner-patient risk communication, it is of academic and practical value to investigate and elucidate the framework and substance of CVD risk communication, aiming to furnish practitioners and their intended risk populations with a standardized template.

Prospect theory also emphasizes that individuals may exhibit risk aversion or risk seeking behavior depending on the framing of health information (32). Message framing is a health communication strategy supported by theoretical foundations aimed at encouraging behavior change by presenting information in either positive or negative terms (33, 34). The manner in which information is framed can influence decision-making outcomes (35). Gain-framed messages focus on the benefits individuals would gain from adopting recommended behaviors, while loss-framed messages emphasize the negative consequences of not engaging in positive behaviors or continuing with negative behaviors.

Previous reviews (36–38) indicated that framed messages have a modest yet reliable impact on altering health behavior. However, conflicting findings have emerged regarding the comparative benefits of positive/gain- versus negative/loss-framed messages (33). For example, loss-framed messages have been confirmed to persuade people to adopt cancer detection behaviors (39) and diabetes self-care (40), while gained-framed messages have received considerable empirical support for physical activity and dental hygiene behaviors (37). In addition, the literature on message framing consistently demonstrated that when a behavior or characteristic of the individual is not taken into account, there is no discernible advantage for gain- or loss-framed messages (41). Scholars have suggested that the potential moderators may affect the advantage in magnitude and orientation (42), such as risk perception (32, 43) or the nature of behavior (44). Consequently, it is imperative to prioritize the clarification of the potential interaction between message framing and risk perception on health behavior. Regrettably, only a limited number of intervention studies have been conducted to investigate the impact of message framing on behaviors related to CVD prevention, especially targeted populations with risk perception subcategorizes.

## 2 Objective

This pilot study aims to clarify the optimal risk communication strategy by examining the compatibility between message framing (gain-framed, loss-framed, or no-framed) and risk perception subgroups (under-perceived vs. over-perceived vs. correct-perceived CVD risk), using a multi-center randomized controlled trial design in Zhejiang province, China. The specific objectives are as follows:

1. To evaluate the effect of message framing on CVD prevention behaviors among individuals with moderate and high CVD risk.
2. To design a series of CVD risk communication messages based on message framing, then to implement the intervention program among individuals with different risk perception subgroups in the communities.
3. To determine the optimal risk communication message for CVD risk populations with risk perception categories to achieve better CVD prevention behavior change, continuum maintenance, and risk reduction.

## 3 Materials and methods

### 3.1 Study design

**Figure 1:**
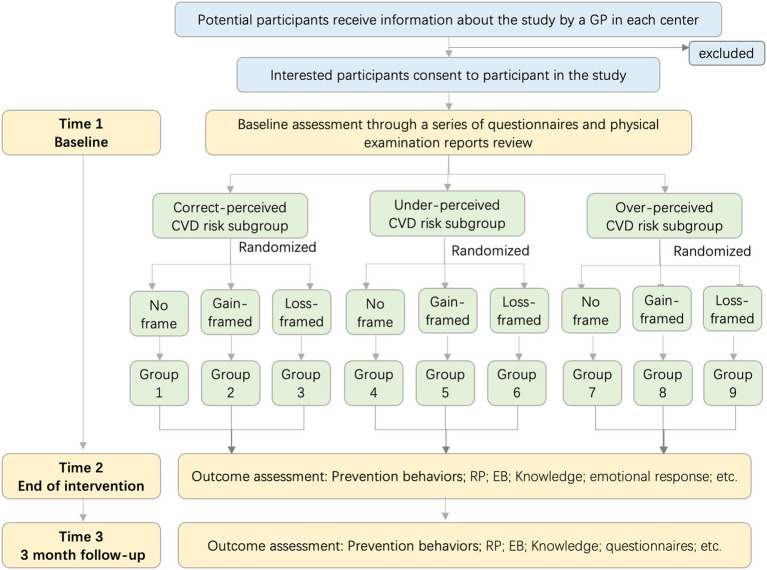
Study flow chart.

A multi-center 3 × 3 randomized factorial, observer-blinded experimental design was conducted to evaluate the effect of gain-, loss-versus no frame message and under-, over- versus correct risk perception. This trial will be implemented on the basis of National Basic Public Health Service Program (45) and Zhejiang Province Basic Public Service standards (2021 edition). Those includes essential health services for all citizens and chronic health management for hypertensions and diabetes, such as having a free physical examination opportunity once a year, and the electronic physical examination recorded in the community health care system, so that general practitioners (GP) could provide continuous health management services. This study was approved by the Human Research Ethics Committee of the second affiliated Hospital of Zhejiang University school of Medicine (No.2023-0877). It was registered and approved by China Clinical Trials Center (ChiCTR2300076337). We followed the SPIRIT guidelines, and all participants will be followed up for 3 months (Figure 1).

### 3.2 Study setting

This study will be conducted in three communities located in Hangzhou, Zhejiang Province. The health-related cultural norms and values, as well as the availability of local resources for adopting healthier lifestyles and accessing convenient medication consultation, differ between urban and rural communities. Therefore, including urban and rural sites will enhance the generalizability of the findings and facilitate result dissemination across diverse locations. As mentioned before, these risk populations had a free physical examination once a year according to the chronic disease management standard of Zhejiang Province. Thus, biochemical indicators (total cholesterol, high density lipoprotein cholesterol, etc.) were obtained from the health examination recording, eliminating the need for additional blood samples in this study. All study sites will follow the same study procedures.

### 3.3 Blinding

Due to the inherent characteristics of the intervention, both researchers who responsible for intervention and participants will not be blinded to the group allocations. The initial collection of baseline data commenced first, after which participants will be informed of their allocated group. Two other well-trained researchers, who were blinded to group allocation, will be responsible for data collection. The investigators responsible for implementing the intervention will not participate in the data collection process. The statistician conducting the subsequent data analysis will be blinded to the group allocation.

### 3.4 Participants and recruitment

#### 3.4.1 Eligibility criteria

1. Inclusion criteria: Aged 20–80 years old; permanent residence; accessible for reliable CVD risk calculation indicators from health examination records (waistline, blood pressure, total cholesterol, high density lipoprotein cholesterol measured in last 6 months); a 10-year risk of absolute CVD risk (based on China-PAR model) of at least 5%; had access to a telephone to receive picture or video message from WeChat; normal visual acuity and hearing (with correction); speaking with Mandarin; be able to adhere to all study procedures.
2. Exclusion criteria: Experienced incident CVD or stroke before; experienced severe anxiety; engagement in concurrent participation in another health research study; current or planned pregnancy during the study period; restricted physical activity due to other medical conditions; and ongoing treatment for severe disease or terminal medical condition.

#### 3.4.2 Sample size

Previous risk communication intervention studies were utilized to estimate the sample size. In our previous survey (46), we observed a baseline proportion of healthy lifestyle adherence at approximately 30% among participants with familiar sample characteristics. For the intervention groups, we anticipate a relative increase of at least 50% (47), with an expected difference of 25% between the gain- and loss-framed groups (39). This corresponds to an estimated effect size (W) for Chi-square of 0.20 (48), aligning closely with recommendations from prior studies (38). To achieve a power of 80%, an alpha error rate of 0.05, a standard deviation of 5%, and an estimated effect size (W) of 0.20, a total of 198 participants will be required for our primary outcome, which measures a healthier lifestyle encompassing physical activity, diet, and prevention medication at 3 months. These participants will be evenly distributed into three groups: gain-framed, loss-framed, and no-frame message interventions. However, considering the dropout rate of 20%, a sample size of 240 participants will be required.

#### 3.4.3 Recruitment

A total of 240 participants will be recruited at the chronic disease management clinic of each community between October and November 2023. In order to screen eligibility participants effectively, the research team will collaborate with community clinic staff to review medical records of individuals with hypertension, diabetes, or dyslipidemia. The 10-year CVD absolute risk will be estimated individually using the China-PAR model (Prediction for Atherosclerotic cardiovascular disease Risk) through an online calculator.^1^ Participants whose risk values exceed 5% will be marked as potentially eligible participants. Patients who meet the eligibility criteria will be contacted by the GP to gage their interest in learning more about this research project. Subsequently, proficient study personnel will reach out to the participants via telephone by the investigators within a two-week timeframe to provide detailed information about the study. Eligible participants will be recruited after signed consent is obtained. Each participant will be assigned a unique identification code, such as JA001. The initial character of the code signifies the study site (e.g., J = Jinhua), the subsequent character represents the risk perception group (A = Under-perceived, B = Over-perceived, C = correct-perceived), and the final three numerical digits correspond to the sequence of enrollment. This code will be used for the purpose of randomization.

#### 3.4.4 Risk perception categories

The risk perception categories (correct perceived, under-perceived or over-perceived CVD risk) are determined by comparing risk perception level (CVD risk perception assessed using ABCD-C) (49) and 10-year CVD risk level (10-year CVD absolute risk calculated using China-PAR) (50, 51) with cross-tabulation using the baseline data, and the details were described in our previous study (19).

### 3.5 Randomization and allocation concealment

The researcher, who is not involved in the recruitment stage using R software to pre-generate a randomization list, which determined participant’s allocation to each intervention groups: arm 1(no frame message), arm 2 (gain-framed message) or arm 3 (loss-framed message). Subsequently, sealed envelopes containing group assignments were prepared by an independent researcher. Upon participant enrollment and completion of baseline assessments, an authorized team member opened the sealed envelope corresponding to each participant’s unique identification code to determine their group assignment. Furthermore, participants will not be informed of their group allocation until they provide written consent.

### 3.6 The intervention procedure

The cardiovascular risk communication information will be delivered as standard health message about individual’s CVD risk and risk coping which not contain any particular message framing (arm 1), gain-framed message (arm 2) and loss-framed message (arm 3). Participants will be given the opportunity to withdraw at any point during the procedure.

#### 3.6.1 Arm1: CVD risk communication based on no-framed message (control)

The CVD risk communication content will be derived from the Chinese CVD primary prevention guidelines (2023), CVD risk assessment and management guidelines (2019), Chinese guideline on healthy lifestyle to prevent CVD (2020), comprehensive prevention and treatment guidelines of CVD for community population (2020), Chinese guideline for lipid management (2023), et al., which includes two core message elements: “*why CVD risk is important?*” and “*why CVD risk is relevant to you?*.” To be more specific, the first message element includes the definition and etiology of CVD, the importance of CVD risk management; and the second message element includes *Your* CVD risk factors, risk value, risk effect and risk coping. The message elements and contents have been reviewed by 16 experts from the field of Cardiology (*n* = 3), general medicine (*n* = 3), CVD nursing (*n* = 4), community nursing (*n* = 3), public health (*n* = 1), health education and health promotion (*n* = 1), information communication (*n* = 1). Details of the intervention procedure can be found in Supplementary Table S2.

A total of 48 videos will be designed according to those risk communication contents, with 16 videos allocated to each intervention arm. Each video has a duration of 60 to 90 s to achieve optimal communication efficacy (52). The intervention will span a duration of 8 weeks, with participants receiving two sessions per week. These video sessions will be sent on Sunday and Wednesday morning between 7:00 and 9:00 a.m. weekly. This schedule will be tailored to the participants’ reading habits and working regulations to ensure the content does not adversely affect their work and sleep. Those videos will be transmitted to each participant through the mobile communication software WeChat. Subsequent to each video, participants will be prompted to respond two specific questions pertaining to the most notable keywords, as well as a central question regarding the content of the video. Their responses will ascertain that they have viewed the video carefully and comprehended its content thoroughly. In order to promote participant compliance, previous research suggests the utilization of financial incentives (47). As per the study protocol, participants who successfully answer both questions will be granted a reward of 1 RMB. After intervention procedure finished, all participants will receive a report of the CVD risk assessment and communication to further read and think aloud if necessary. Two weeks prior to the scheduled follow-up, participants will be sent a message containing details regarding the study procedure, as well as a reminder for the forthcoming 3-month follow-up.

#### 3.6.2 Arm 2: CVD risk communication based on gain-framed message

Positive framing differed only in message presentation (40), the content of information was equivalent with the control group. The message will adopt a gain-framed approach, emphasizing the potential positive outcomes associated with a correct understanding of CVD risk and adherence to appropriate risk management behaviors. For instance, in the module titled ‘Your CVD risk value’, the statement provided to the gain-framed group will state that their risk of developing CVD within the next 10 years is 6.3%. This value exceeds the recommended ideal risk level of 4.4% (with the ideal level of modifiable risk factors). Consequently, out of a group of 100 men with the same age and laboratory results, it is projected that 6 individuals will develop CVD within the next decade. Additionally, two individuals will be exempt from CVD due to effective control of ideal risk factors. This information will be accompanied by two highlighted pictograms illustrating the potential benefits. In the module of ‘weight control’, the gain-framed group will be presented with the following statement: “Maintaining a healthy weight and waist circumference can be advantageous in controlling blood pressure, glucose, and lipid levels, thereby reducing the risk of CVD. Specifically, a reduction of 1 cm in waist circumference can lead to 1.48 times decrease in the 10-year CVD risk.” This statement will be accompanied by an illustrative image depicting individuals with healthy weight and waist circumference. The frequency and timing of video delivery will align with that of the control group.

#### 3.6.3 Arm 3: CVD risk communication based on loss-framed message

Negative framing differed only in message presentation (40), and the information will focus on the adverse consequences that arise from a lack of proper understanding of CVD risk and the neglect of preventive behaviors.

For instance, in the module titled “Your CVD risk value,” the statement provided to the loss-framed group will state that their risk of developing CVD within the next 10 years is 6.3%, which exceeds the desired risk level of 4.4%. This indicates that out of a sample of 100 men with the same age and laboratory results, approximately 6 individuals will experience CVD within the next decade. Furthermore, an additional two individuals are expected to develop CVD within the same timeframe due to inadequate control of risk factors. This information will be accompanied by two highlighted pictograms illustrating the potential losses. In the module focused on weight control, the loss-framed group will be presented with the following statement: “Individuals who are overweight and have a higher waist circumference may experience greater difficulty in managing blood pressure, glucose levels, and lipid levels, consequently increasing their risk of cardiovascular disease. Specifically, a 1 cm increase in waist circumference is associated with a 1.48 times higher 10-year cardiovascular disease risk.” This information will be accompanied by a visual representation of obesity, particularly central abdominal obesity. The frequency and timing of video delivery will align with those in the control group.

### 3.7 Outcomes and measurements

Two well-trained independent investigators, who are unaware of the group allocation, will conduct the outcome assessment in the chronic disease management clinic of each community. Baseline assessments will include demographic factors such as age, gender, marital status, education level, ethnic group, employment status, monthly income, subjective numerical ability (53) and medical history (hypertension/diabetes/dyslipidemia).

#### 3.7.1 Primary outcomes

The primary outcome aims to assess two key aspects: (1) CVD prevention behaviors, encompassing healthy physical activity, a balanced diet, and adherence to preventative medication; and (2) CVD absolute risk.

Healthy physical activity will be assessed using the questionnaire of self-reported International Physical Activity Questionnaire-short version (IPAQ) (54). The responses were processed and aggregated using the IPAQ guidelines for Chinese (55). Participants who achieved a minimum of 150 min of moderate-intensity physical activities or 75 min of vigorous-intensity physical activities per week were considered to have fulfilled the criteria for adequate physical activity (56).

Healthy diet will be assessed through healthy diet score based on the updated Chinese Dietary Guideline (57). The assessment included the weekly consumption of six food groups, namely fresh fruit, fresh vegetables, whole grains, fish and other seafood (consumed more than once per week), bean and bean food (consumed at least four times per week), and red meat (consumed less than seven times per week). The response that met the established criteria received a score of 1 for each food group, and the cumulative score was calculated (with a maximum score of 6). The healthy group was defined as individuals with a total score of 4 or higher (58).

Taking preventative medication will be evaluated through medical prescription for stain/lipid lowering, anticoagulants, antihypertension, glucose-lowering medications (59), based on data obtained from the community physician health check system.

The estimation of CVD absolute risk, including both 10-year and lifetime CVD risk, will be performed using the China-PAR equation (50). Participants will be classified into three groups based on the China-PAR cut-off value specified in the Chinese guidelines (56), namely low risk (<5%), moderate risk (5–9.9%) and high risk (≥10%); for lifetime CVD risk: low risk (<32.8%), and high risk (≥32.8%).

#### 3.7.2 Secondary outcomes

Secondary outcomes include CVD risk perception, efficacy belief, CVD related knowledge, major adverse cardiovascular events (MACE), the emotional response, physical indicators (Blood pressure, blood glucose, waist circumference and BMI) and other lifestyle related risk factors [smoking, drinking status, and subjective health status (60)].

CVD risk perception, efficacy belief and CVD related knowledge will be evaluated using the Chinese version of Attitude and Beliefs about Cardiovascular Disease Risk Questionnaire (ABCD-C) (49), which comprises 26 items across four dimensions. Efficacy belief will be assessed using the item 19, 20, 22, and 24, following the recommendation by Rimal and Juon (61). This scale has demonstrated good validity and reliability in both its original and Chinese versions, as evidenced by Cronbach’s α values ranging from 0.70 to 0.94. The emotional response will be assessed using two items on a 10-point scale (1–10; with a total range of 2–20), which have been adopted from the existing literature (62). An example of such item is “How concerned are you by reading this information?.” MACEs are defined as the composite endpoints of cardiovascular death, spontaneous myocardial infraction, and target vessel revascularization at 3 months follow up (63). The smoking and drinking status, as well as physical indicators, were extracted from the follow-up records of participants by GPs.

#### 3.7.3 Other measurements

To assess the manipulation check, participants were asked to rate the extent to which they believed the video messages emphasized the advantages of engaging in CVD risk management or the disadvantages of not doing so. This rating was measured on a scale of 1 to 7, with 1 indicating a greater emphasis on the benefits, 4 indicating an equal focus on both benefits and risks, and 7 indicating a greater emphasis on the disadvantages (34). Participants will be deemed to have passed the manipulation check if they choose the item that aligns with their assigned message condition (64). The level of engagement will be evaluated based on the responses to questions following each video message, with a threshold of more than 75% of responses indicating high engagement (35). The qualitative components of risk communication experience after intervention procedure finished at 8 weeks will be evaluated using semi-structured interviews with the participants. This approach enables us to investigate participants’ experiences and delve into the social, cultural, and environmental factors that may impact their responses to the intervention. Such exploration is invaluable for interpreting quantitative findings accurately and enhancing intervention program effectiveness through iterative improvements. Details of the interview outline can be found in Supplementary Table S2. Study participants will be randomly selected at each study site among enrolled samples. The interviews will be stopped once data saturation is reached (Table 1).

**Table 1:** Research activities.

	Baseline	Post assessment	Follow-up assessment
	T0	T1	T2
Informed consent	√		
Demographic, socioeconomic assessment	√		
CVD risk factors assessment	√	√	√
10-year CVD risk calculation	√		√
Lifetime CVD risk calculation	√		√
Physical activity assessment (self-reported)	√	√	√
Healthy diet assessment (self-reported)	√	√	√
Medication prescription	√	√	√
CVD related knowledge	√	√	√
CVD risk perception	√	√	√
Health related efficacy	√	√	√
Subjective health status	√	√	√
Major adverse cardiovascular events (MACE)			√
Emotional response		√	
Manipulation check		√	
Participants engagement		√	
Interviews with participants		√	

### 3.8 Statistical analysis

#### 3.8.1 Data analysis

Descriptive statistical analysis will be conducted for all measurements. The distributions of categorical baseline variables will be compared using χ^2^ test among the three intervention groups. The difference in continuous variables will be analyzed using either one-way analysis of variance (ANOVA) or a non-parametric rank-sum test. The differential changes in the primary (i.e., healthy behaviors) and secondary (i.e., risk perception) outcomes at T1 and T2, relative to T0, across the three groups will be assessed using generalized estimating equations (GEE) models. GEE methods were applied to estimate model parameters using a binomial distribution for the variance function, a logit link function, and accounting for clustering via an assumed exchangeable working correlation structure (65). The outcomes will be used as dependent variables (one for each model) and, as independent variables, the intervention arms (no-framed, gain-framed, loss-framed message), the factors time (T0, T1, T2), the covariates (risk perception categories) and their corresponding interaction term. The other potential confounders (i.e., variables with baseline imbalances, study sites) will be added as independent variables to adjust GEE model. An exchangeable working correlation structure will be assumed in order to assess the association between factors and outcomes. The statistical significance of each parameter in the model will be analyzed using a Wald test. To account for the potential occurrence of type 1 errors, a Bonferroni correction will be implemented for all GEE models, with a *p*-value threshold of less than 0.003 indicating statistically significant differences (66). The final model’s results will be presented as estimated odds ratio (*OR*) and 95% confidence interval (*CI*) for each significant prognostic variable. We will also conduct the sensitivity analysis to assess the non-response mechanism (67) and an attrition analysis (68) to identify the differential dropout rates and dropout by group interaction on sociodemographic and pretest variables that may pose a threat the equity of the findings (69). Subgroup analysis was performed for cases that did not pass the manipulation check. The principle of “intention to treat (ITT)” will be applied in conducting all primary and secondary outcome analyses. Moreover, the patterns of missingness would be recorded across variables and time points to provide insight into non-random missingness. Statistical significance will be determined by results with *p* < 0.05 in two-sided tests. The analysis will be carried out using SPSS software for MAC (version 26.0) and R software (version 4.3.1).

The interview transcripts will be recorded, double transcribed, checked, and entered into NVIVO version 11 for analysis. Content analysis framework approach will be employed to analyze the qualitative data. To minimize potential bias, two experienced researchers will independently code the data. The identified concepts will be grouped into categories and themes.

#### 3.8.2 Missing data plan

Although we aim to retain the majority of participants, missing data is inevitable when subjects withdraw for reasons beyond our control. To avoid the missing data, the researcher will systematically examine each post-video question to identify instances where subjects have not been filled and subsequently issue reminders. Besides, we set the outcome assessments on site, and the researchers will check the questionnaire to remind participants to fill in the missing data immediately. In addition, the physical examination data will be extracted from medical recordings of the community health care system to minimize missing data. The GEE model will be employed due to its ability to generate unbiased estimates, even when missing data is present, assuming the missing is completely random. Reasons for non-adherence and non-retention will be recorded.

#### 3.8.3 Data quality control

To ensure the dependability of data collection, all researchers across the study sites will undergo standardized training on the study protocol and quality control techniques for data collection. The data will be anonymized and stored on a secure server that enables immediate updates and maintains confidentiality. Only the principal investigator can access to the master copy of the data. Access and utilization of the anonymized data will be restricted solely to authorized members of the research team. In addition, it is critical to avoid contamination to ensure data accuracy. The intervention messages will be conveyed through WeChat software, it is possible that the participants from the same community might discuss these contents. To minimize contamination, participants will be instructed not to send, share or exchange those messages with others until the conclusion of study. If necessary, we will resort to contamination-adjusted ITT analysis using instrumental variables analysis (70).

## 4 Discussion

A number of evidence-based guidelines exist for CVD primary prevention (56, 71). however, effectively incorporating risk-based treatment paradigms into clinical practice necessitates the implementation of strategies that accurately convey risk information to individuals (72). We systematically designed CVD risk communication message from the message element, subjects and outlines based on related guidelines and expert’s consultation to ensure the reliability. While the use of visual aids, charts, and protocols has been suggested to facilitate discussions about CVD risk, it is evident that a standardized approach may not be suitable for all individuals (73). This discrepancy in risk information preferences underscores the significance of tailoring risk communication to accommodate variations in risk perception. Therefore, we stratified the risk population into distinct subgroups based on a comparison of their risk perception and objective risk level. Subsequently, the risk communication message will be delivered using gain-, loss-, or no-framed formats. Finally, an experimental study design will be employed to ascertain the most effective matching strategy between risk perception subgroups and risk information format. To the best of our knowledge, this will be the inaugural RCT to establish the optimal health communication strategies that consider both individuals’ risk characteristics and information preference in the context of CVD risk management. This research holds significant value for primary CVD practice in China and other resource-limited regions.

CVD risk communication was not only risk assessment and risk value disclosure, but also about the risk coping guidance. Previous studies have demonstrated the importance of engaging in a conversation regarding individual CVD risk between patients and healthcare professionals in order to maximize the impact of the risk information provided (74). However, effectively delivering comprehensive and consistent risk communication to a large population at risk of CVD poses a significant challenge for healthcare providers and the healthcare system, particularly in China (75). The scarcity of primary healthcare providers further complicates the task of dedicating sufficient time to conduct detailed and in-depth health communication. Our intervention study has the potential to offer health providers in community or clinic settings with dependable and efficient health communication information for the purpose of carrying out CVD risk management. This intervention study can provide health providers in community or clinic with reliable and effective health communication information to conduct CVD risk management. Based on recent research results, we will further develop CVD risk communication software that intelligently aligns risk perception categories with optimal risk communication information, thereby significantly enhancing CVD primary prevention practice.

Several systematic reviews have reported contradictory results, failing to favor either loss-framed or gain-framed messages for specific behaviors (36, 37). Scholars have advised that moderators, including the subjective meaning individuals assign to risk, should be tested (44). According to prospect theory (24), the perceived risk associated with the recommended behavior determines the relative persuasiveness of gain- and loss-framed messages. Given the significance of CVD risk reduction, there exists a theoretical rationale for investigating the influence of risk perception and message framing effects on CVD preventive behaviors. Risk perception is a multifaceted construct that encompasses subjective evaluations influenced by cultural and individual value systems (28). Misconceptions or inaccurate attitudes toward risk can greatly affect how people respond to it (18). For instance, someone who underestimates the dangers might engage in risky activities without precautions. Regrettably, previous investigations (43) have predominantly concentrated on the variable of risk perception without assessing its accuracy—a crucial aspect that might elucidate the modest impact of framed message, thus, addressing this gap in the literature is imperative. Furthermore, cultural and value attributes can influence not only how risks are perceived but also how they are communicated and managed (28). Effective risk communication strategies must consider cultural differences to reach diverse audiences effectively. Thus, it is valuable to figure out the potential interaction between message framing and risk perception on health behavior in non-western cultural context. Significantly, our study aims to delineate the optimal matching strategy between risk perception categories and framework information, providing an empirical foundation for individuals with diverse perception characteristics to personalize risk communication and decision-making support. This tailored approach holds promise for healthcare personnel in enhancing the management of cardiovascular diseases more effectively. Building upon this work, future investigations can refine the content of framed message to align with local cultural nuances and the values of the target audience. Additionally, exploring optimal matching pattern across varied cultural backgrounds will yield invaluable insights for further mitigating the global burden of cardiovascular disease.

However, it is important to acknowledge several limitations associated with this pilot study. First of all, the assessment of outcomes was conducted over a relatively brief period based on practical reasons, necessitating further investigation into the long-term effectiveness of the intervention. Secondly, our intervention will be implemented online through smartphone (WeChat App), potentially introducing selection bias. However, there were more than 1.3 billion WeChat active users worldwide in 2023 (76) and more than one-third of individuals regularly obtained health information by WeChat in China (77), WeChat has been increasingly used for disseminating health information. In addition, the representativeness of samples was improved in our study through enrolled participants from urban and rural areas, as well as different educational level. Third, the utilization of self-report forms to assess physical activity and diet may be susceptible to recall bias. Subsequent research will explore the adoption of accelerometers (78) or image recognition technique (79) to detect the actual status of lifestyle changing. However, we did conduct objective measurements such as blood pressure, blood glucose, total cholesterol, high-density lipoprotein, etc. at the stage of pre- and post-intervention, and calculated the 10-year risk of CVD. Comparing changes in these measures also offers a way to reflect the impact of lifestyle modifications. Finally, our study was conducted in several communities within a city in southeast China, yet the sample remains non-representative of the entire population. Given the significance of cultural nuances in health communication, further research is warranted to determine the substantial impact of message framing on the improvement of risk perception and modifiable behaviors across various regions in China.

## Ethics statement

The studies involving humans were approved by The Institutional Review Board of the second affiliated hospital of Zhejiang University School of Medicine. The studies were conducted in accordance with the local legislation and institutional requirements. The participants provided their written informed consent to participate in this study.

## Author contributions

ZG: Conceptualization, Methodology, Writing – original draft. QW: Data curation, Investigation, Resources, Writing – review & editing. XiW: Conceptualization, Methodology, Writing – review & editing. YD: Data curation, Resources, Writing – review & editing. YM: Data curation, Investigation, Writing – review & editing. YQ: Methodology, Writing – review & editing. YZ: Conceptualization, Formal Analysis, Methodology, Project administration, Writing – review & editing. XuW: Data curation, Methodology, Writing – review & editing. JJ: Conceptualization, Funding acquisition, Methodology, Supervision, Writing – review & editing.

## Footnotes

^1^ https://www.cvdrisk.com.cn/ASCVD/Eval
