# Supplementary material for: Effects of message framing and risk perception on health communication for optimum cardiovascular disease primary prevention: a protocol for a multicenter randomized controlled study
Source: Front Public Health. 2024 Mar 14;12:1308745. doi: 10.3389/fpubh.2024.1308745 (PMC10972929; doi:10.3389/fpubh.2024.1308745)
Supplement: Supplementary file 1 [file Data_Sheet_1.docx]

**Table S1 Theories description included in this study**

| **Theory** | **Description** | **Elements** | **Relevant Studies** | **Tips to Current Research** |
| --- | --- | --- | --- | --- |
| Health Belief Model (HBM) | Predicts health-related behaviors based on perceived threats, benefits, barriers, and self-efficacy. | Perceived threats, benefits, barriers, self-efficacy | Glanz et al. (2015) - Examined the application of the HBM in promoting physical activity among adults.  Champion et al. (2008) - Explored the use of HBM in promoting colorectal cancer screening among adults. | Perceived threats, benefits and self-efficacy are core element of risk perception assessment. |
| Risk Perception Attitude framework (RPA) | Emphasizes the role of perceived risk and attitudes in shaping behavior towards health threats. | Perceived risk, attitudes, behavior | Ferrer and Klein (2015) - Examined the influence of risk perceptions on cancer screening behaviors among adults.  Slovic et al. (2005) - Investigated the role of affect and risk perception in decision making regarding environmental hazards. | Perceived risk could affect health behavior, which is the theoretical basis of our study. |
| Protection Motivation Theory (PMT) | Focuses on how individuals assess threats and coping mechanisms | Threat appraisal, coping appraisal, motivation | Floyd et al. (2000) - Investigated the application of PMT in understanding HIV preventive behaviors among college students.  Milne et al. (2000) - Examined the role of PMT in understanding sunscreen use and sun protection behaviors. | How could risk perception affect risk coping mechanism, which explored with specific context in our study. |

**Reference**

1. Glanz K, Rimer BK, Viswanath K. Health behavior: Theory, research, and practice. John Wiley & Sons; 2015.

2. Champion VL, Skinner CS, Menon U, Rawl S, Giesler RB, Monahan P, et al. A breast cancer fear scale: psychometric development. Journal of Health Psychology. 2004 Nov;9(6):753-62.

3. Ferrer RA, Klein WM. Risk perceptions and health behavior. Current Opinion in Psychology. 2015 Oct 1;5:85-9.

4. Slovic P, Finucane ML, Peters E, MacGregor DG. The affect heuristic. European Journal of Operational Research. 2007 Dec 16;177(3):1333-52.

5. Floyd DL, Prentice-Dunn S, Rogers RW. A meta-analysis of research on protection motivation theory. Journal of Applied Social Psychology. 2000 Sep;30(2):407-29.

6. Milne S, Sheeran P, Orbell S. Prediction and intervention in health‐related behavior: A meta‐analytic review of protection motivation theory. Journal of Applied Social Psychology. 2000 Feb;30(1):106-43.

**Table S2 Schedule of health communication**

| **Week** | **No.** | **Message element** | **Subjects** | **Outline** | **Arm 1**  **(Control)** | **Arm 2** | **Arm 3** |
| --- | --- | --- | --- | --- | --- | --- | --- |
| 1 | 1 | ***Why*** CVD risk is important? | Etiology of CVD | Etiology of CVD, signs and symptoms | No frame | Gain-famed | Loss-framed |
|  | 2 |  | CVD risk management | Risk factors of CVD and the importance of CVD risk management | No frame | Gain-famed | Loss-framed |
| 2 | 3 | ***Why*** CVD risk is relevant to you? | Your CVD risk source | Interpret the origin of your CVD risk | No frame | Gain-famed | Loss-framed |
|  | 4 |  | Your CVD risk value | Calculate and interpret your 10-year CVD risk/ lifetime CVD risk | No frame | Gain-famed | Loss-framed |
| 3 | 5 |  | Your CVD risk effect | The effect to your health (disease/complication/the quality of life) | No frame | Gain-famed | Loss-framed |
|  | 6 |  |  | The effect to your life (job/family/income) | No frame | Gain-famed | Loss-framed |
| 4 | 7 |  | Your CVD risk coping | Healthy lifestyle- diet nutrition- diet pattern and healthy food selection | No frame | Gain-famed | Loss-framed |
|  | 8 |  |  | Healthy lifestyle- diet nutrition- Cooking methods | No frame | Gain-famed | Loss-framed |
| 5 | 9 |  |  | Healthy lifestyle- physical activity- Types | No frame | Gain-famed | Loss-framed |
|  | 10 |  |  | Healthy lifestyle- physical activity- Intensity and Frequency | No frame | Gain-famed | Loss-framed |
| 6 | 11 |  |  | Healthy lifestyle- Minimize Sedentary Behavior | No frame | Gain-famed | Loss-framed |
|  | 12 |  |  | Healthy lifestyle- Weight control/ Smoking cessation/ Limit drinking (select individually) | No frame | Gain-famed | Loss-framed |
| 7 | 13 |  |  | Healthy lifestyle- Mentally healthy | No frame | Gain-famed | Loss-framed |
|  | 14 |  |  | Healthy lifestyle- Sleep healthy | No frame | Gain-famed | Loss-framed |
| 8 | 15 |  |  | Prevention medication- Medication plan and goals | No frame | Gain-famed | Loss-framed |
|  | 16 |  |  | Prevention medication- Medication monitoring and adherence | No frame | Gain-famed | Loss-framed |

**Table S3 Interview outline**

| **No.** | **Outline** |
| --- | --- |
| 1 | Please provide a detailed experience pertaining to the intervention process. |
| 2 | Could you elaborate on the current state of your lifestyle?  What notable changes have occurred since the intervention? |
| 3 | Please expound upon your medication adherence at present.  What notable changes have occurred since the intervention? |
| 4 | What aspect of the intervention left the most profound impression on you? Why? |
| 5 | In your opinion, what factors contributed the most to your progress?  Why do you believe this to be the case?  Additionally, could you identify any aspects of the intervention that were less effective and explain your reasoning? |
| 6 | How do you respond when viewing these videos?  Furthermore, what influence do you believe this information has had on your behavior?  What is the underlying cause of this effect? |
| 7 | Do you possess any comments or suggestions regarding the intervention program? Additionally, could you identify any areas that require further improvement? |
